# One step at a time: use of single-session rTMS to test novel targets for substance use disorders

**DOI:** 10.1038/s41386-025-02149-5

**Published:** 2025-06-06

**Authors:** Heather Burrell Ward

**Affiliations:** Department of Psychiatry and Behavioral Sciences, Vanderbilt University Medical Center, Nashville, TN USA

**Keywords:** Addiction, Translational research

Neuromodulation has emerged as a promising potential treatment for substance use disorders (SUDs). Meta-analyses have demonstrated that neuromodulation, including repetitive transcranial magnetic stimulation (rTMS), reduces substance use, craving, and withdrawal across a range of SUDs [1].

However, the underlying mechanism of these behavioral effects has not been elucidated. This may be because many existing studies of rTMS for SUDs have tested weeks-long interventions without neurobiological outcomes. Many clinical trials of rTMS have bypassed the fundamental research that would first uncover the basic mechanism of neuromodulation’s neural and behavioral effects. As a result, the field has been left searching for the underlying mechanism of rTMS’s effects on SUDs, which is a major obstacle for future studies.

To date, the overwhelming majority of studies of rTMS for SUDs have targeted the left dorsolateral prefrontal cortex (DLPFC). Although experts acknowledge the need for novel targets, few have actually ventured beyond the DLPFC.

In this issue, Petersen et al. use single sessions of rTMS to uncover the basic mechanism of rTMS effects on nicotine craving, withdrawal, and negative affect [2]. To do this, they use an elegant, within-subjects crossover design (*n* = 72) with pre- and post-intervention neuroimaging to disentangle effects of rTMS (10 Hz, 100% resting motor threshold) on connectivity and behavior.

Petersen et al. tackle a novel neurobiological mechanism driving SUDs. It is well-known that people with SUDs have disruptions in the connectivity of many canonical resting-state networks, including the default mode network (DMN), executive control network (ECN), and salience network (SN). It is also known that while rTMS provides focal stimulation, its effects propagate across resting-state networks.

In this paper, rather than applying rTMS to one-size-fits-all anatomical targets, Petersen et al. compare four different personalized, *network-based* targets to test if modulating network connectivity affects craving, withdrawal, and negative affect. They compare the following four targets as nodes of each of their respective resting-state networks: (1) DLPFC (ECN); (2) superior frontal gyrus (SFG, SN); (3) posterior parietal cortex (PPC, DMN); and (4) v5 (control target).

The findings of this experiment are unexpected: SFG outperforms the other targets, reducing both craving and withdrawal. Yet, SFG stimulation did not change connectivity within or between the SN, DMN, or ECN. The SFG target was selected based on maximal connectivity with the insula, a core SN region. Overall, the SFG shows promise for future studies of multiple rTMS sessions.

But why didn’t SFG stimulation affect whole SN connectivity? There are a few possible explanations. First, it is possible that, rather than modulating connectivity of the entire network, SFG stimulation did in fact affect connectivity of select SN connectivity edges, which Petersen et al. have not yet investigated. Second, it is possible that a single session of SFG stimulation was not sufficient to change the connectivity of the entire SN, which may require multiple stimulation sessions. The absence of a whole-network effect is consistent with our own previous research comparing single and multiple sessions of network-targeted rTMS for nicotine use, where a single session of rTMS was insufficient to modulate average connectivity of the entire network, but multiple sessions did change whole-network connectivity (Ward et al., *In Preparation*). Therefore, it is possible that single and multi-session rTMS interventions will have dose-dependent neural effects – a single session might affect one particular network edge while multiple sessions may produce a more widespread, network-wide effect. Moreover, rTMS operates by inducing synaptic plasticity [3], so the rTMS-induced alterations in functional connectivity may vary when assessed minutes, hours, days, weeks, or even months after an intervention. Multiple rTMS sessions and a longer timescale to assessment may allow for the detection of robust, network-wide effects.

Petersen et al. also observed main effects of DLPFC stimulation on craving, although the target x time interaction was not significant. DLPFC stimulation increased connectivity in the somatomotor, default mode, and dorsal attention networks but not in the ECN. Interestingly, PPC stimulation did not affect behavior or connectivity, despite previous evidence linking craving to DMN connectivity.

Another novel aspect of this study relates to the state dependence of rTMS effects. It is well-known that rTMS effects vary based on brain state [4]. In this study, participants arrived with at least 12 hours of abstinence from nicotine. Few existing studies have applied rTMS in the setting of acute abstinence from tobacco, so the effects of an acute withdrawal state on behavioral and neural effects are yet unknown. In the study that led to the U.S. Food and Drug Administration clearance of rTMS for smoking cessation, rTMS was applied for the two weeks leading up to a quit date [5]. Future studies will need to study the effects of brain state on response to rTMS for SUDs.

One of the major strengths of this study is its systematic interrogation of multiple active rTMS targets using a within-subjects approach. This type of study design is powerful and highly efficient and sets the stage for subsequent trials of multiple rTMS sessions to these targets, which will ultimately progress to smoking cessation interventions. This approach is exactly what the field of rTMS for SUDs needs at this moment. By using a methodical, stepwise approach to testing rTMS interventions and their neurobiological effects, we can accumulate enough evidence for well-supported large clinical trials of rTMS for SUDs.

This study is also novel in its use of personalized targets. Although rTMS for depression has moved to personalized, connectivity-based targets, the SUD field has lagged until now. In the future, we would not only use personalized targets but also use precision medicine approaches to identify prospectively which patients would benefit from which rTMS stimulation target and protocol. Petersen et al. also take steps toward this goal in their examination of sex differences. They find that men have twice the response to SFG stimulation compared to women – an interesting finding for future exploration.

In summary, these studies – using single rTMS sessions to probe the mechanism of rTMS for SUDs, are critical stepping stones that line the path toward highly effective neuromodulation interventions for SUDs.
